# Crystal structure of (*E*)-4-(acet­oxy­imino)-*N*-allyl-3-isopropyl-2,6-di­phenyl­piperi­dine-1-carbo­thio­amide

**DOI:** 10.1107/S2056989015012499

**Published:** 2015-07-04

**Authors:** T. Mohandas, K. Gokula Krishnan, S. Balamurugan, William T. A. Harrison, V. Thanikachalam, P. Sakthivel

**Affiliations:** a Department of Physics, Shri Angalamman College of Engineering and Technology, Siruganoor, Tiruchirappalli, India; b Department of Chemistry, Annamalai University, Annamalainagar, Chidambaram, India; c Department of Physics, Rover College of Engineering and Technology, Perambalur, India; d Department of Chemistry, University of Aberdeen, Aberdeen AB24 3UE, Scotland; e Department of Physics, Urumu Dhanalakshmi College, Tiruchirappalli, India

**Keywords:** crystal structure, carbo­thio­amide, piperidine, acet­oxy­imino, inversion dimers

## Abstract

The title compound, C_26_H_31_N_3_O_2_S, crystallizes with two mol­ecules (*A* and *B*) in the asymmetric unit. In each case, the piperidine ring exists in a twist-boat conformation. The dihedral angle between the phenyl rings is 46.16 (12)° in mol­ecule *A* and 44.95 (12)° in mol­ecule *B*. In both mol­ecules, the allyl side chain is disordered over two orientations in a 0.649 (9):0.351 (9) ratio for mol­ecule *A* and 0.826 (10):0.174 (10) ratio for mol­ecule *B*. In the crystal, neither mol­ecule forms a hydrogen bond from its N—H group, presumably due to steric hindrance. *A*+*A* and *B*+*B* inversion dimers are formed, linked by pairs of weak C—H⋯O hydrogen bonds enclosing *R*_2_^2^(22) ring motifs.

## Related literature   

For the structural properties and biological potentials of carbothio­amides, see: Wilkerson *et al.* (1996 ▸); Koca *et al.* (2013 ▸); Liu *et al.* (2012 ▸); Malik *et al.*(2013 ▸). For related structures, see: Park *et al.* (2012*a* ▸,*b* ▸).
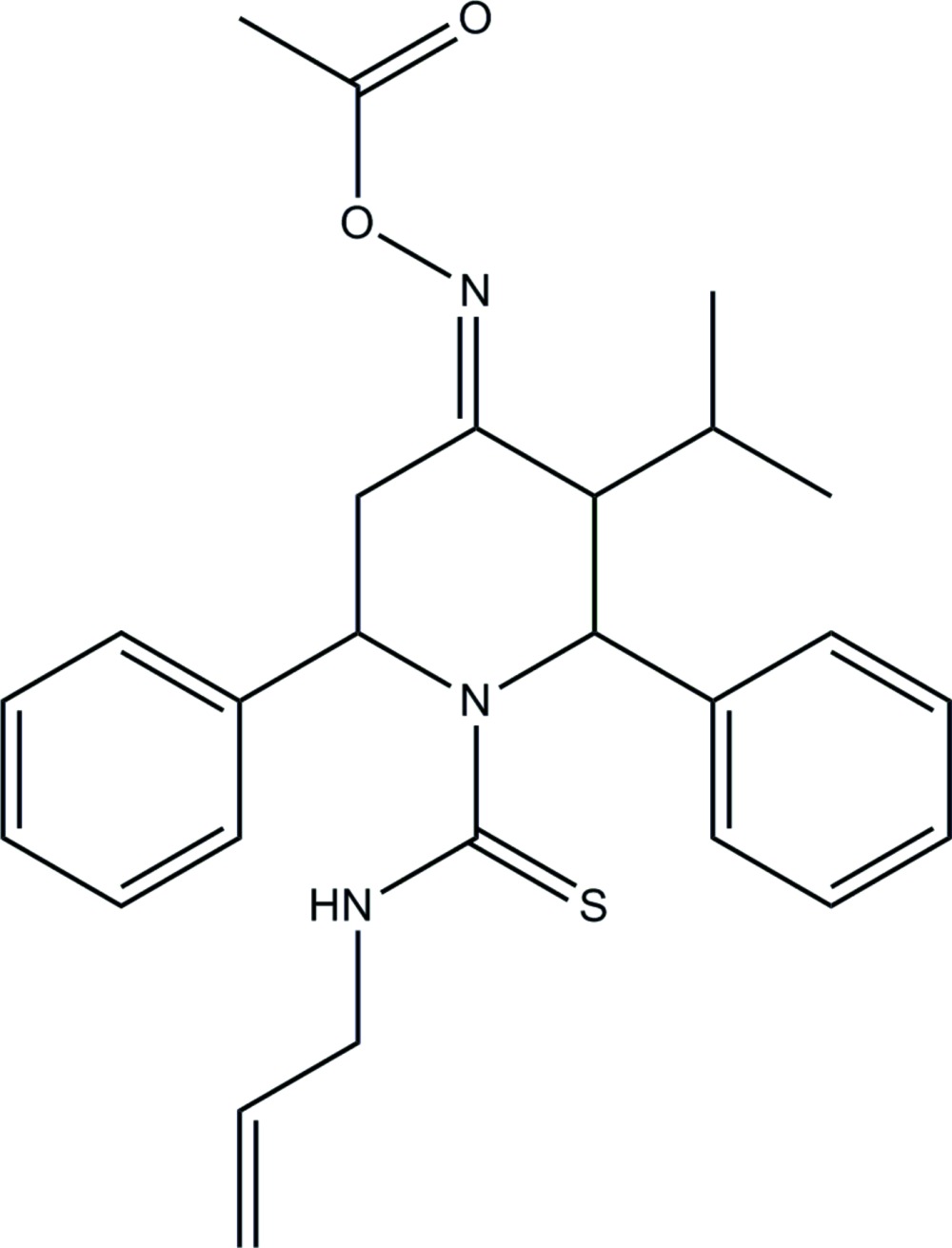


## Experimental   

### Crystal data  

C_26_H_31_N_3_O_2_S
*M* *_r_* = 449.60
Triclinic, 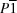

*a* = 12.0434 (6) Å
*b* = 14.0479 (7) Å
*c* = 15.2740 (7) Å
α = 82.161 (2)°
β = 72.463 (2)°
γ = 80.094 (2)°
*V* = 2417.4 (2) Å^3^
*Z* = 4
Mo *K*α radiation
μ = 0.16 mm^−1^
*T* = 293 K
0.28 × 0.25 × 0.24 mm

### Data collection  

Bruker Kappa APEXII CCD diffractometer
Absorption correction: multi-scan (*SADABS*; Bruker, 2008 ▸) *T*_min_ = 0.956, *T*_max_ = 0.962
74637 measured reflections
11303 independent reflections
6065 reflections with *I* > 2σ(*I*)
*R*_int_ = 0.050

### Refinement  

*R*[*F* ^2^ > 2σ(*F* ^2^)] = 0.049
*wR*(*F* ^2^) = 0.146
*S* = 1.01
11303 reflections
627 parameters
93 restraints
H-atom parameters constrained
Δρ_max_ = 0.19 e Å^−3^
Δρ_min_ = −0.24 e Å^−3^

### 

Data collection: *APEX2* (Bruker, 2008 ▸); cell refinement: *SAINT* (Bruker, 2008 ▸); data reduction: *SAINT*; program(s) used to solve structure: *SHELXS97* (Sheldrick, 2008 ▸); program(s) used to refine structure: *SHELXL97* (Sheldrick, 2008 ▸); molecular graphics: *ORTEP-3 for Windows* (Farrugia, 2012 ▸) and *Mercury* (Macrae *et al.*, 2008 ▸); software used to prepare material for publication: *SHELXL97* and *PLATON* (Spek, 2009 ▸).

## Supplementary Material

Crystal structure: contains datablock(s) global, I. DOI: 10.1107/S2056989015012499/su5159sup1.cif

Structure factors: contains datablock(s) I. DOI: 10.1107/S2056989015012499/su5159Isup2.hkl

Supporting information file. DOI: 10.1107/S2056989015012499/su5159Isup3.cml

**Fig. 1.**
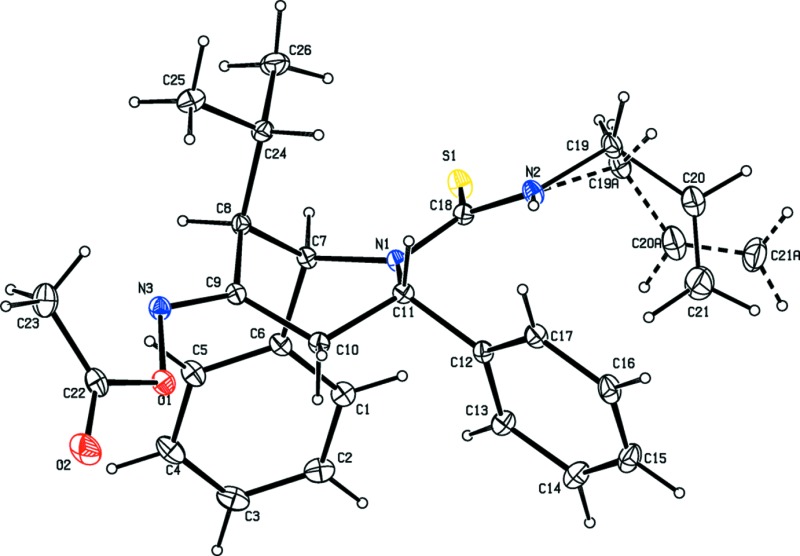
The molecular structure of molecule A of the title compound, with atom labelling. Displacement ellipsoids are drawn at the 30% probability level.

. DOI: 10.1107/S2056989015012499/su5159fig1.tif
The mol­ecular structure of mol­ecule A of the title compound, with atom labelling. Displacement ellipsoids are drawn at the 30% probability level.

**Fig. 2.**
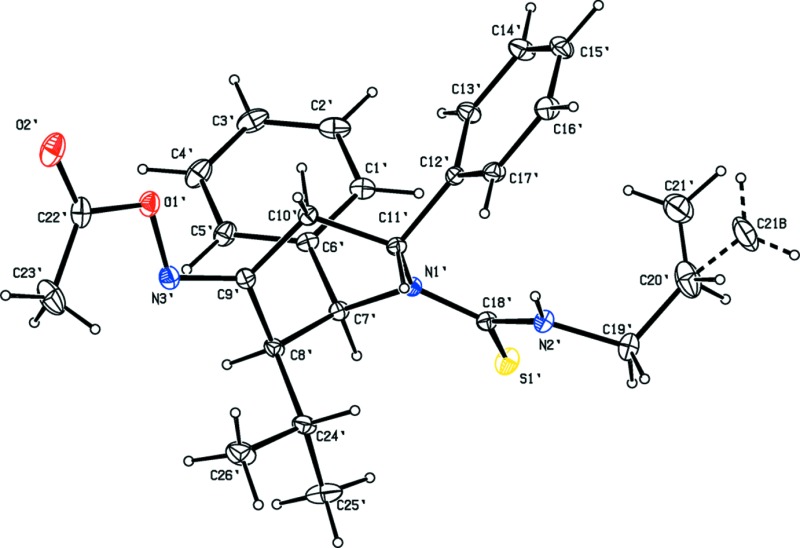
The molecular structure of molecule B of the title compound, with atom labelling. Displacement ellipsoids are drawn at the 30% probability level.

. DOI: 10.1107/S2056989015012499/su5159fig2.tif
The mol­ecular structure of mol­ecule B of the title compound, with atom labelling. Displacement ellipsoids are drawn at the 30% probability level.

**Fig. 3.**
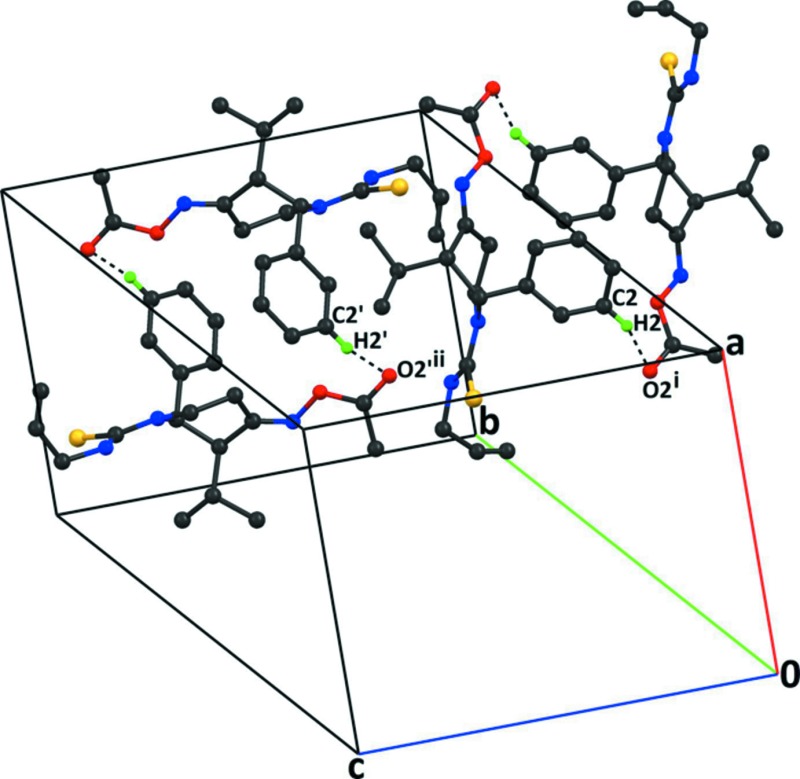
A partial view of the crystal packing of the title compound, with the C—H···O hydrogen bonds shown as dashed lines (see Table 1 for details). Other H atoms and the minor components of the allyl groups have been omitted for clarity.

. DOI: 10.1107/S2056989015012499/su5159fig3.tif
A partial view of the crystal packing of the title compound, with the C—H⋯O hydrogen bonds shown as dashed lines (see Table 1 for details). Other H atoms and the minor components of the allyl groups have been omitted for clarity.

CCDC reference: 1024973

Additional supporting information:  crystallographic information; 3D view; checkCIF report

## Figures and Tables

**Table 1:** Hydrogen-bond geometry (, )

*D*H*A*	*D*H	H*A*	*D* *A*	*D*H*A*
C2H2O2^i^	0.93	2.59	3.298(4)	133
C2H2O2^ii^	0.93	2.59	3.332(4)	137

Symmetry codes: (i) 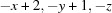; (ii) 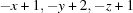.

## supplementary crystallographic information

### S1. Structural commentary

The chemistry of carbo­thio­amide derivatives are much explored today because of their structural properties and their biological potential as anti-HIV (Wilkerson *et al.*, 1996), anti­cancer (Koca *et al.*, 2013), anti­tubercular (Liu *et al.*2012), and anti­convulsant (Malik *et al.*, 2013) agents.

The bond distances and bond angles in the two independent molecules (A and B) of the title compound, Fig. 1, agree well with those reported for closely related compounds (Park *et al.*, 2012a,b). In each molecule the piperidine ring exists in a twist-boat conformation with the puckering parameters Q = 0.7236, θ = 96.90 (19) and π = 267.97 (19) Å in A and Q = 0.7245, θ = 82.9 (2) and π = 87.38 (19) Å in B. The dihedral angle between the phenyl rings is 46.13 (13) ° in molecule A and 44.97 (13)° in molecule B.

In the crystal, the individual molecules form A—A and B—B inversion dimers with R_2_^2^(22) ring motifs (Table 1 and Fig. 2).

### S2. Synthesis and crystallization

To a solution of 3-iso­propyl-2,6-di­phenyl­piperidin-4-one *O*-acetyl oxime (0.5 g, 1.5 mmol) in dry DCM (5 ml), pyridine (1.5 eq) and allyl­iso­thio­cyanate (0.17 g, 1.75 mmol) were added drop wise over 5 min to a 50 ml Erlenmeyer flask. The reaction mixture was subjected to ultrasound irradiation for 1 h at ambient temperature and the progress of the reaction was monitored by TLC. Upon completion of the reaction, the mixture was slowly poured into crushed ice giving the crude product as a precipitate. It was subjected to recrystallization from absolute ethanol giving the title compound in good yield (0.62 g, 76%), as colourless block-like crystals.

### S3. Refinement

Crystal data, data collection and structure refinement details are summarized in Table 2. The positions of the hydrogen atoms bound to the N and C atoms were identified from difference electron density maps. The NH H atoms were refined with *U*_iso_(H) = 1.2U_eq_(N). The C-bound H atoms were refined as riding atoms: C—H = 0.93 - 0.98 Å with *U*_iso_(H) = 1.5*U*_eq(_C) for methyl H atoms and 1.2U_eq_(C) for other H atoms. The atoms of the allyl groups (C19, C20 and C21) are disordered over two orientations in a 0.649 (9):0.351 (9) ratio for molecule A, and a 0.826 (10):0.174 (10) ratio for atom C21' in molecule B. They were modelled with restrained bonds and angles based on the average values found for a non-disordered allyl group.

### Crystal data

**Table:**

C_26_H_31_N_3_O_2_S	*Z* = 4
*M**_r_* = 449.60	*F*(000) = 960
Triclinic, *P*1	*D*_x_ = 1.235 Mg m^−^^3^
Hall symbol: -P 1	Mo *K*α radiation, λ = 0.71073 Å
*a* = 12.0434 (6) Å	Cell parameters from 6065 reflections
*b* = 14.0479 (7) Å	θ = 1.5–27.8°
*c* = 15.2740 (7) Å	µ = 0.16 mm^−^^1^
α = 82.161 (2)°	*T* = 293 K
β = 72.463 (2)°	Block, colourless
γ = 80.094 (2)°	0.28 × 0.25 × 0.24 mm
*V* = 2417.4 (2) Å^3^	

### Data collection

**Table:**

Bruker Kappa APEXII CCD diffractometer	11303 independent reflections
Radiation source: fine-focus sealed tube	6065 reflections with *I* > 2σ(*I*)
Graphite monochromator	*R*_int_ = 0.050
ω & φ scans	θ_max_ = 27.8°, θ_min_ = 1.5°
Absorption correction: multi-scan (*SADABS*; Bruker, 2008)	*h* = −15→15
*T*_min_ = 0.956, *T*_max_ = 0.962	*k* = −18→18
74637 measured reflections	*l* = −19→19

### Refinement

**Table:**

Refinement on *F*^2^	Primary atom site location: structure-invariant direct methods
Least-squares matrix: full	Secondary atom site location: difference Fourier map
*R*[*F*^2^ > 2σ(*F*^2^)] = 0.049	Hydrogen site location: mixed
*wR*(*F*^2^) = 0.146	H-atom parameters constrained
*S* = 1.01	*w* = 1/[σ^2^(*F*_o_^2^) + (0.0578*P*)^2^ + 0.7614*P*] where *P* = (*F*_o_^2^ + 2*F*_c_^2^)/3
11303 reflections	(Δ/σ)_max_ = 0.001
627 parameters	Δρ_max_ = 0.19 e Å^−^^3^
93 restraints	Δρ_min_ = −0.24 e Å^−^^3^

### Special details

**Table:**

Geometry. All esds (except the esd in the dihedral angle between two l.s. planes) are estimated using the full covariance matrix. The cell esds are taken into account individually in the estimation of esds in distances, angles and torsion angles; correlations between esds in cell parameters are only used when they are defined by crystal symmetry. An approximate (isotropic) treatment of cell esds is used for estimating esds involving l.s. planes.
Refinement. Refinement of *F*^2^ against ALL reflections. The weighted *R*-factor *wR* and goodness of fit *S* are based on *F*^2^, conventional *R*-factors *R* are based on *F*, with *F* set to zero for negative *F*^2^. The threshold expression of *F*^2^ > σ(*F*^2^) is used only for calculating *R*-factors(gt) *etc*. and is not relevant to the choice of reflections for refinement. *R*-factors based on *F*^2^ are statistically about twice as large as those based on *F*, and *R*- factors based on ALL data will be even larger.

### Fractional atomic coordinates and isotropic or equivalent isotropic displacement parameters (Å^2^)

**Table:**

	*x*	*y*	*z*	*U*_iso_*/*U*_eq_	Occ. (<1)
C1	0.9104 (2)	0.32104 (16)	0.16483 (16)	0.0505 (6)	
H1	0.8287	0.3293	0.1823	0.061*	
C2	0.9726 (3)	0.25361 (18)	0.10359 (18)	0.0634 (7)	
H2	0.9324	0.2168	0.0804	0.076*	
C3	1.0929 (3)	0.24062 (19)	0.07682 (18)	0.0679 (8)	
H3	1.1347	0.1957	0.0350	0.082*	
C4	1.1506 (2)	0.29384 (19)	0.11184 (19)	0.0666 (8)	
H4	1.2324	0.2848	0.0942	0.080*	
C5	1.0898 (2)	0.36120 (17)	0.17328 (17)	0.0546 (6)	
H5	1.1309	0.3968	0.1967	0.065*	
C6	0.96801 (19)	0.37627 (15)	0.20040 (14)	0.0414 (5)	
C7	0.89823 (17)	0.44607 (15)	0.27223 (14)	0.0392 (5)	
H7	0.8777	0.4069	0.3315	0.047*	
C8	0.96601 (18)	0.52344 (15)	0.28429 (14)	0.0398 (5)	
H8	1.0386	0.4899	0.2971	0.048*	
C9	1.00053 (18)	0.58243 (14)	0.19289 (14)	0.0394 (5)	
C10	0.91588 (18)	0.59728 (15)	0.13722 (14)	0.0410 (5)	
H10C	0.9416	0.5509	0.0911	0.049*	
H10D	0.9167	0.6618	0.1052	0.049*	
C11	0.78954 (17)	0.58595 (14)	0.19439 (13)	0.0375 (5)	
H11	0.7606	0.6397	0.2338	0.045*	
C12	0.71303 (18)	0.59542 (15)	0.13008 (14)	0.0400 (5)	
C13	0.7181 (2)	0.52194 (17)	0.07673 (16)	0.0521 (6)	
H13	0.7691	0.4647	0.0799	0.063*	
C14	0.6473 (2)	0.5336 (2)	0.01873 (18)	0.0650 (7)	
H14	0.6509	0.4839	−0.0168	0.078*	
C15	0.5724 (2)	0.6170 (2)	0.01290 (19)	0.0692 (8)	
H15	0.5240	0.6235	−0.0255	0.083*	
C16	0.5684 (2)	0.6912 (2)	0.06366 (19)	0.0648 (7)	
H16	0.5184	0.7487	0.0588	0.078*	
C17	0.6389 (2)	0.68088 (17)	0.12239 (16)	0.0514 (6)	
H17	0.6362	0.7316	0.1566	0.062*	
C18	0.68301 (18)	0.46797 (15)	0.31362 (14)	0.0403 (5)	
C19	0.4679 (7)	0.5156 (14)	0.3736 (6)	0.055 (2)	0.649 (9)
H19A	0.4322	0.5710	0.4089	0.066*	0.649 (9)
H19B	0.4741	0.4591	0.4171	0.066*	0.649 (9)
C20	0.3887 (7)	0.5005 (5)	0.3198 (4)	0.073 (2)	0.649 (9)
H20A	0.3097	0.5017	0.3523	0.088*	0.649 (9)
C21	0.4150 (8)	0.4867 (6)	0.2373 (4)	0.107 (3)	0.649 (9)
H21A	0.4926	0.4847	0.2011	0.129*	0.649 (9)
H21B	0.3570	0.4782	0.2115	0.129*	0.649 (9)
C19A	0.4658 (12)	0.509 (2)	0.3549 (12)	0.051 (4)	0.351 (9)
H19C	0.4126	0.5685	0.3711	0.061*	0.351 (9)
H19D	0.4597	0.4656	0.4106	0.061*	0.351 (9)
C20A	0.4303 (10)	0.4633 (11)	0.2866 (13)	0.080 (4)	0.351 (9)
H20C	0.4864	0.4176	0.2531	0.096*	0.351 (9)
C21A	0.3336 (10)	0.4799 (8)	0.2700 (9)	0.094 (4)	0.351 (9)
H21C	0.2744	0.5250	0.3017	0.113*	0.351 (9)
H21D	0.3205	0.4471	0.2259	0.113*	0.351 (9)
C22	1.2103 (2)	0.71539 (19)	0.0584 (2)	0.0602 (7)	
C23	1.2410 (3)	0.7428 (2)	0.1369 (2)	0.0812 (9)	
H23A	1.2808	0.6869	0.1642	0.122*	
H23B	1.1707	0.7672	0.1819	0.122*	
H23C	1.2916	0.7921	0.1157	0.122*	
C24	0.9001 (2)	0.58617 (17)	0.36463 (15)	0.0495 (6)	
H24	0.8309	0.6245	0.3499	0.059*	
C25	0.9766 (2)	0.65575 (19)	0.37572 (18)	0.0647 (7)	
H25A	1.0489	0.6200	0.3838	0.097*	
H25B	0.9362	0.6907	0.4287	0.097*	
H25C	0.9934	0.7007	0.3217	0.097*	
C26	0.8579 (3)	0.5235 (2)	0.45415 (17)	0.0723 (8)	
H26A	0.9227	0.4780	0.4649	0.108*	
H26B	0.7978	0.4890	0.4499	0.108*	
H26C	0.8267	0.5640	0.5042	0.108*	
N1	0.78573 (14)	0.49402 (11)	0.25558 (11)	0.0373 (4)	
N2	0.58584 (15)	0.53170 (13)	0.31824 (13)	0.0453 (5)	
H2A	0.590 (2)	0.5882 (13)	0.2876 (15)	0.054*	
N3	1.10177 (16)	0.60888 (13)	0.16899 (12)	0.0471 (5)	
S1	0.67667 (6)	0.35980 (4)	0.37701 (5)	0.05813 (19)	
O1	1.12691 (13)	0.65613 (12)	0.07647 (11)	0.0552 (4)	
O2	1.25123 (18)	0.74278 (16)	−0.02053 (15)	0.0901 (7)	
C1'	0.6908 (2)	0.82063 (16)	0.39025 (18)	0.0530 (6)	
H1'	0.6887	0.8289	0.3293	0.064*	
C2'	0.6264 (2)	0.75462 (18)	0.4523 (2)	0.0675 (8)	
H2'	0.5812	0.7192	0.4328	0.081*	
C3'	0.6292 (3)	0.7414 (2)	0.5417 (2)	0.0747 (9)	
H3'	0.5852	0.6976	0.5833	0.090*	
C4'	0.6960 (3)	0.7921 (2)	0.56967 (19)	0.0746 (8)	
H4'	0.6988	0.7822	0.6305	0.090*	
C5'	0.7602 (2)	0.85858 (17)	0.50906 (16)	0.0579 (7)	
H5'	0.8054	0.8931	0.5296	0.069*	
C6'	0.75794 (18)	0.87427 (14)	0.41829 (15)	0.0418 (5)	
C7'	0.83309 (19)	0.94263 (15)	0.34869 (14)	0.0407 (5)	
H7'	0.9052	0.9025	0.3168	0.049*	
C8'	0.87150 (18)	1.01997 (15)	0.39090 (15)	0.0430 (5)	
H8'	0.9098	0.9862	0.4369	0.052*	
C9'	0.76054 (19)	1.08015 (15)	0.44174 (15)	0.0413 (5)	
C10'	0.66016 (18)	1.09535 (15)	0.40147 (14)	0.0418 (5)	
H10A	0.6182	1.1601	0.4117	0.050*	
H10B	0.6064	1.0495	0.4334	0.050*	
C11'	0.69701 (17)	1.08337 (14)	0.29862 (14)	0.0376 (5)	
H11'	0.7406	1.1369	0.2666	0.045*	
C12'	0.58795 (18)	1.09288 (15)	0.26641 (13)	0.0387 (5)	
C13'	0.5171 (2)	1.02040 (17)	0.28710 (17)	0.0523 (6)	
H13'	0.5363	0.9635	0.3215	0.063*	
C14'	0.4187 (2)	1.0319 (2)	0.2571 (2)	0.0654 (7)	
H14'	0.3718	0.9827	0.2715	0.078*	
C15'	0.3888 (2)	1.1149 (2)	0.20649 (19)	0.0663 (7)	
H15'	0.3228	1.1217	0.1854	0.080*	
C16'	0.4564 (2)	1.1878 (2)	0.18714 (18)	0.0644 (7)	
H16'	0.4356	1.2450	0.1537	0.077*	
C17'	0.5557 (2)	1.17704 (17)	0.21699 (15)	0.0513 (6)	
H17'	0.6012	1.2272	0.2035	0.062*	
C18'	0.82442 (18)	0.96574 (15)	0.18875 (15)	0.0426 (5)	
C19'	0.8314 (2)	1.0136 (2)	0.02547 (16)	0.0624 (7)	
H19E	0.8962	0.9610	0.0135	0.075*	
H19F	0.8592	1.0713	−0.0113	0.075*	
C20'	0.7370 (4)	0.9896 (3)	−0.0056 (3)	0.1015 (12)	
H20E	0.7519	0.9936	−0.0693	0.122*	0.826 (10)
H20F	0.7353	0.9249	0.0174	0.122*	0.174 (10)
C21'	0.6441 (5)	0.9659 (5)	0.0372 (5)	0.130 (3)	0.826 (10)
H21E	0.6225	0.9602	0.1013	0.156*	0.826 (10)
H21F	0.5931	0.9530	0.0064	0.156*	0.826 (10)
C21B	0.6593 (18)	1.003 (2)	−0.0433 (18)	0.111 (9)	0.174 (10)
H21G	0.6414	1.0634	−0.0730	0.133*	0.174 (10)
H21H	0.6178	0.9527	−0.0424	0.133*	0.174 (10)
C22'	0.6484 (3)	1.21816 (19)	0.6224 (2)	0.0672 (8)	
C23'	0.7637 (3)	1.2480 (3)	0.6141 (3)	0.1040 (12)	
H23D	0.7529	1.2970	0.6551	0.156*	
H23E	0.7973	1.2735	0.5518	0.156*	
H23F	0.8154	1.1928	0.6299	0.156*	
C24'	0.9607 (2)	1.07945 (18)	0.32045 (17)	0.0546 (6)	
H24'	0.9267	1.1084	0.2706	0.066*	
C25'	1.0733 (3)	1.0147 (2)	0.2795 (3)	0.0981 (11)	
H25D	1.1302	1.0535	0.2399	0.147*	
H25E	1.0581	0.9693	0.2443	0.147*	
H25F	1.1033	0.9800	0.3281	0.147*	
C26'	0.9864 (3)	1.1606 (2)	0.3641 (2)	0.0795 (9)	
H26D	1.0106	1.1343	0.4179	0.119*	
H26E	0.9168	1.2069	0.3813	0.119*	
H26F	1.0481	1.1920	0.3207	0.119*	
N1'	0.77728 (14)	0.99094 (12)	0.27709 (11)	0.0382 (4)	
N2'	0.80050 (17)	1.03032 (14)	0.12168 (12)	0.0464 (5)	
H2B	0.7510 (18)	1.0837 (13)	0.1341 (16)	0.056*	
N3'	0.76056 (18)	1.10743 (14)	0.51771 (13)	0.0514 (5)	
S1'	0.90805 (6)	0.85825 (5)	0.16427 (5)	0.0636 (2)	
O1'	0.64564 (15)	1.15591 (12)	0.56286 (11)	0.0583 (4)	
O2'	0.5553 (2)	1.24742 (16)	0.67412 (15)	0.0951 (7)	

### Atomic displacement parameters (Å^2^)

**Table:**

	*U*^11^	*U*^22^	*U*^33^	*U*^12^	*U*^13^	*U*^23^
C1	0.0547 (15)	0.0396 (13)	0.0555 (14)	−0.0050 (11)	−0.0158 (12)	−0.0004 (11)
C2	0.088 (2)	0.0427 (14)	0.0590 (16)	−0.0050 (14)	−0.0216 (15)	−0.0058 (12)
C3	0.086 (2)	0.0431 (15)	0.0578 (16)	0.0035 (14)	−0.0022 (15)	−0.0052 (12)
C4	0.0585 (17)	0.0486 (15)	0.0714 (18)	0.0025 (13)	0.0057 (14)	−0.0008 (13)
C5	0.0480 (14)	0.0438 (13)	0.0638 (16)	−0.0055 (11)	−0.0050 (12)	−0.0031 (12)
C6	0.0415 (12)	0.0338 (11)	0.0417 (12)	−0.0031 (9)	−0.0059 (10)	0.0050 (9)
C7	0.0349 (11)	0.0383 (11)	0.0395 (11)	−0.0034 (9)	−0.0082 (9)	0.0054 (9)
C8	0.0346 (11)	0.0421 (12)	0.0419 (12)	−0.0029 (9)	−0.0131 (9)	0.0005 (9)
C9	0.0368 (12)	0.0357 (11)	0.0448 (12)	−0.0053 (9)	−0.0092 (10)	−0.0056 (9)
C10	0.0421 (12)	0.0394 (12)	0.0401 (12)	−0.0113 (10)	−0.0103 (10)	0.0049 (9)
C11	0.0417 (12)	0.0317 (11)	0.0370 (11)	−0.0052 (9)	−0.0108 (9)	0.0032 (9)
C12	0.0372 (12)	0.0403 (12)	0.0396 (12)	−0.0087 (9)	−0.0096 (9)	0.0076 (9)
C13	0.0595 (15)	0.0462 (14)	0.0549 (14)	−0.0103 (11)	−0.0240 (12)	0.0026 (11)
C14	0.0779 (19)	0.0685 (18)	0.0614 (16)	−0.0261 (15)	−0.0349 (15)	0.0058 (14)
C15	0.0659 (18)	0.089 (2)	0.0623 (17)	−0.0299 (17)	−0.0349 (15)	0.0215 (16)
C16	0.0457 (15)	0.0737 (19)	0.0645 (17)	0.0002 (13)	−0.0165 (13)	0.0205 (15)
C17	0.0465 (14)	0.0527 (14)	0.0488 (14)	−0.0010 (11)	−0.0122 (11)	0.0061 (11)
C18	0.0397 (12)	0.0403 (12)	0.0406 (12)	−0.0073 (10)	−0.0117 (10)	0.0002 (9)
C19	0.037 (3)	0.066 (5)	0.054 (4)	−0.012 (3)	−0.005 (2)	0.007 (3)
C20	0.051 (4)	0.082 (4)	0.083 (4)	−0.012 (3)	−0.013 (3)	−0.001 (3)
C21	0.088 (6)	0.156 (6)	0.080 (5)	−0.053 (4)	−0.022 (4)	0.025 (4)
C19A	0.039 (6)	0.047 (6)	0.059 (7)	−0.001 (5)	−0.009 (5)	0.004 (6)
C20A	0.049 (6)	0.090 (7)	0.091 (8)	−0.012 (5)	−0.012 (6)	0.009 (7)
C21A	0.066 (7)	0.134 (9)	0.099 (8)	−0.039 (6)	−0.037 (7)	−0.001 (6)
C22	0.0421 (14)	0.0560 (15)	0.0750 (19)	−0.0132 (12)	−0.0036 (13)	−0.0016 (14)
C23	0.0684 (19)	0.076 (2)	0.106 (2)	−0.0324 (16)	−0.0274 (18)	−0.0004 (18)
C24	0.0475 (14)	0.0577 (14)	0.0440 (13)	0.0017 (11)	−0.0181 (11)	−0.0066 (11)
C25	0.082 (2)	0.0597 (16)	0.0582 (16)	−0.0085 (14)	−0.0262 (14)	−0.0105 (13)
C26	0.083 (2)	0.090 (2)	0.0461 (15)	−0.0248 (17)	−0.0142 (14)	−0.0060 (14)
N1	0.0339 (9)	0.0341 (9)	0.0401 (9)	−0.0045 (7)	−0.0079 (8)	0.0038 (7)
N2	0.0362 (10)	0.0399 (10)	0.0519 (11)	−0.0051 (8)	−0.0049 (8)	0.0056 (9)
N3	0.0432 (11)	0.0488 (11)	0.0482 (11)	−0.0119 (9)	−0.0106 (9)	0.0017 (9)
S1	0.0500 (4)	0.0464 (4)	0.0679 (4)	−0.0117 (3)	−0.0092 (3)	0.0180 (3)
O1	0.0488 (10)	0.0606 (10)	0.0535 (10)	−0.0221 (8)	−0.0063 (8)	0.0031 (8)
O2	0.0786 (14)	0.0983 (16)	0.0796 (14)	−0.0437 (12)	0.0075 (11)	0.0099 (12)
C1'	0.0465 (14)	0.0403 (13)	0.0686 (16)	−0.0029 (11)	−0.0137 (12)	−0.0027 (11)
C2'	0.0510 (16)	0.0415 (14)	0.103 (2)	−0.0102 (12)	−0.0095 (15)	−0.0049 (14)
C3'	0.0719 (19)	0.0481 (16)	0.081 (2)	−0.0151 (14)	0.0111 (16)	0.0051 (15)
C4'	0.098 (2)	0.0552 (16)	0.0550 (16)	−0.0142 (16)	−0.0009 (15)	0.0055 (13)
C5'	0.0719 (18)	0.0496 (14)	0.0477 (14)	−0.0159 (13)	−0.0100 (12)	0.0041 (11)
C6'	0.0385 (12)	0.0332 (11)	0.0475 (13)	−0.0010 (9)	−0.0066 (10)	−0.0008 (9)
C7'	0.0386 (12)	0.0395 (12)	0.0406 (12)	−0.0016 (9)	−0.0112 (10)	0.0024 (9)
C8'	0.0409 (12)	0.0457 (13)	0.0456 (12)	−0.0117 (10)	−0.0191 (10)	0.0078 (10)
C9'	0.0489 (13)	0.0355 (11)	0.0422 (12)	−0.0136 (10)	−0.0163 (10)	0.0040 (9)
C10'	0.0425 (12)	0.0398 (12)	0.0432 (12)	−0.0039 (10)	−0.0122 (10)	−0.0062 (9)
C11'	0.0349 (11)	0.0360 (11)	0.0397 (11)	−0.0048 (9)	−0.0088 (9)	−0.0005 (9)
C12'	0.0367 (12)	0.0424 (12)	0.0350 (11)	−0.0028 (9)	−0.0089 (9)	−0.0026 (9)
C13'	0.0481 (14)	0.0465 (13)	0.0650 (15)	−0.0091 (11)	−0.0236 (12)	0.0052 (11)
C14'	0.0520 (16)	0.0669 (17)	0.0841 (19)	−0.0182 (13)	−0.0278 (14)	0.0013 (15)
C15'	0.0500 (15)	0.087 (2)	0.0677 (17)	−0.0101 (15)	−0.0309 (14)	0.0058 (15)
C16'	0.0554 (16)	0.0728 (18)	0.0589 (16)	−0.0044 (14)	−0.0223 (13)	0.0232 (14)
C17'	0.0439 (13)	0.0552 (14)	0.0501 (14)	−0.0078 (11)	−0.0123 (11)	0.0093 (11)
C18'	0.0393 (12)	0.0427 (12)	0.0438 (13)	−0.0063 (10)	−0.0095 (10)	−0.0013 (10)
C19'	0.0799 (19)	0.0606 (16)	0.0440 (14)	−0.0020 (14)	−0.0168 (13)	−0.0070 (12)
C20'	0.119 (3)	0.099 (3)	0.105 (3)	0.018 (3)	−0.064 (3)	−0.042 (2)
C21'	0.084 (4)	0.188 (6)	0.134 (6)	0.001 (4)	−0.034 (4)	−0.085 (5)
C21B	0.079 (14)	0.18 (2)	0.103 (19)	0.013 (13)	−0.059 (13)	−0.060 (15)
C22'	0.104 (2)	0.0535 (16)	0.0520 (16)	−0.0091 (16)	−0.0336 (17)	−0.0069 (13)
C23'	0.122 (3)	0.087 (2)	0.136 (3)	−0.003 (2)	−0.077 (3)	−0.047 (2)
C24'	0.0420 (13)	0.0625 (15)	0.0613 (15)	−0.0185 (12)	−0.0199 (12)	0.0140 (12)
C25'	0.0522 (18)	0.098 (2)	0.124 (3)	−0.0241 (17)	0.0116 (18)	−0.011 (2)
C26'	0.074 (2)	0.0738 (19)	0.097 (2)	−0.0384 (16)	−0.0282 (17)	0.0160 (17)
N1'	0.0372 (10)	0.0382 (10)	0.0376 (10)	−0.0009 (8)	−0.0109 (8)	−0.0029 (8)
N2'	0.0531 (12)	0.0440 (11)	0.0381 (10)	−0.0011 (9)	−0.0098 (9)	−0.0040 (9)
N3'	0.0611 (13)	0.0496 (11)	0.0488 (12)	−0.0132 (10)	−0.0211 (10)	−0.0031 (9)
S1'	0.0723 (5)	0.0507 (4)	0.0573 (4)	0.0123 (3)	−0.0119 (3)	−0.0111 (3)
O1'	0.0712 (12)	0.0580 (10)	0.0489 (9)	−0.0131 (9)	−0.0154 (9)	−0.0144 (8)
O2'	0.126 (2)	0.0888 (16)	0.0640 (13)	−0.0094 (14)	−0.0098 (13)	−0.0324 (12)

### Geometric parameters (Å, º)

**Table:**

C1—C6	1.380 (3)	N2—H2A	0.866 (15)
C1—C2	1.381 (3)	N3—O1	1.444 (2)
C1—H1	0.9300	C1'—C6'	1.383 (3)
C2—C3	1.369 (4)	C1'—C2'	1.387 (3)
C2—H2	0.9300	C1'—H1'	0.9300
C3—C4	1.357 (4)	C2'—C3'	1.361 (4)
C3—H3	0.9300	C2'—H2'	0.9300
C4—C5	1.379 (3)	C3'—C4'	1.351 (4)
C4—H4	0.9300	C3'—H3'	0.9300
C5—C6	1.385 (3)	C4'—C5'	1.381 (3)
C5—H5	0.9300	C4'—H4'	0.9300
C6—C7	1.525 (3)	C5'—C6'	1.382 (3)
C7—N1	1.484 (2)	C5'—H5'	0.9300
C7—C8	1.527 (3)	C6'—C7'	1.521 (3)
C7—H7	0.9800	C7'—N1'	1.481 (3)
C8—C9	1.504 (3)	C7'—C8'	1.534 (3)
C8—C24	1.543 (3)	C7'—H7'	0.9800
C8—H8	0.9800	C8'—C9'	1.506 (3)
C9—N3	1.268 (3)	C8'—C24'	1.542 (3)
C9—C10	1.485 (3)	C8'—H8'	0.9800
C10—C11	1.532 (3)	C9'—N3'	1.271 (3)
C10—H10C	0.9700	C9'—C10'	1.486 (3)
C10—H10D	0.9700	C10'—C11'	1.523 (3)
C11—N1	1.486 (2)	C10'—H10A	0.9700
C11—C12	1.516 (3)	C10'—H10B	0.9700
C11—H11	0.9800	C11'—N1'	1.490 (3)
C12—C17	1.381 (3)	C11'—C12'	1.515 (3)
C12—C13	1.382 (3)	C11'—H11'	0.9800
C13—C14	1.380 (3)	C12'—C17'	1.376 (3)
C13—H13	0.9300	C12'—C13'	1.383 (3)
C14—C15	1.360 (4)	C13'—C14'	1.371 (3)
C14—H14	0.9300	C13'—H13'	0.9300
C15—C16	1.368 (4)	C14'—C15'	1.366 (4)
C15—H15	0.9300	C14'—H14'	0.9300
C16—C17	1.388 (3)	C15'—C16'	1.363 (4)
C16—H16	0.9300	C15'—H15'	0.9300
C17—H17	0.9300	C16'—C17'	1.381 (3)
C18—N2	1.335 (3)	C16'—H16'	0.9300
C18—N1	1.363 (2)	C17'—H17'	0.9300
C18—S1	1.687 (2)	C18'—N2'	1.338 (3)
C19—N2	1.455 (5)	C18'—N1'	1.367 (3)
C19—C20	1.490 (8)	C18'—S1'	1.685 (2)
C19—H19A	0.9700	C19'—N2'	1.445 (3)
C19—H19B	0.9700	C19'—C20'	1.459 (4)
C20—C21	1.238 (7)	C19'—H19E	0.9700
C20—H20A	0.9300	C19'—H19F	0.9700
C21—H21A	0.9300	C20'—C21'	1.192 (5)
C21—H21B	0.9300	C20'—C21B	1.216 (9)
C19A—N2	1.457 (7)	C20'—H20E	0.9300
C19A—C20A	1.495 (10)	C20'—H20F	0.9300
C19A—H19C	0.9700	C21'—H21E	0.9300
C19A—H19D	0.9700	C21'—H21F	0.9300
C20A—C21A	1.242 (9)	C21B—H21G	0.9300
C20A—H20C	0.9300	C21B—H21H	0.9300
C21A—H21C	0.9300	C22'—O2'	1.203 (3)
C21A—H21D	0.9300	C22'—O1'	1.355 (3)
C22—O2	1.194 (3)	C22'—C23'	1.483 (4)
C22—O1	1.355 (3)	C23'—H23D	0.9600
C22—C23	1.470 (4)	C23'—H23E	0.9600
C23—H23A	0.9600	C23'—H23F	0.9600
C23—H23B	0.9600	C24'—C25'	1.509 (4)
C23—H23C	0.9600	C24'—C26'	1.514 (4)
C24—C25	1.511 (3)	C24'—H24'	0.9800
C24—C26	1.520 (3)	C25'—H25D	0.9600
C24—H24	0.9800	C25'—H25E	0.9600
C25—H25A	0.9600	C25'—H25F	0.9600
C25—H25B	0.9600	C26'—H26D	0.9600
C25—H25C	0.9600	C26'—H26E	0.9600
C26—H26A	0.9600	C26'—H26F	0.9600
C26—H26B	0.9600	N2'—H2B	0.878 (16)
C26—H26C	0.9600	N3'—O1'	1.443 (3)
			
C6—C1—C2	120.9 (2)	C19—N2—H2A	114.8 (17)
C6—C1—H1	119.5	C19A—N2—H2A	113 (2)
C2—C1—H1	119.5	C9—N3—O1	110.22 (17)
C3—C2—C1	120.4 (3)	C22—O1—N3	113.05 (18)
C3—C2—H2	119.8	C6'—C1'—C2'	120.5 (3)
C1—C2—H2	119.8	C6'—C1'—H1'	119.7
C4—C3—C2	119.3 (3)	C2'—C1'—H1'	119.7
C4—C3—H3	120.3	C3'—C2'—C1'	120.4 (3)
C2—C3—H3	120.3	C3'—C2'—H2'	119.8
C3—C4—C5	120.9 (3)	C1'—C2'—H2'	119.8
C3—C4—H4	119.5	C4'—C3'—C2'	119.7 (3)
C5—C4—H4	119.5	C4'—C3'—H3'	120.1
C4—C5—C6	120.6 (2)	C2'—C3'—H3'	120.1
C4—C5—H5	119.7	C3'—C4'—C5'	120.8 (3)
C6—C5—H5	119.7	C3'—C4'—H4'	119.6
C1—C6—C5	117.8 (2)	C5'—C4'—H4'	119.6
C1—C6—C7	120.21 (19)	C4'—C5'—C6'	120.7 (3)
C5—C6—C7	121.8 (2)	C4'—C5'—H5'	119.7
N1—C7—C6	112.82 (17)	C6'—C5'—H5'	119.7
N1—C7—C8	109.01 (16)	C5'—C6'—C1'	117.9 (2)
C6—C7—C8	114.69 (17)	C5'—C6'—C7'	121.8 (2)
N1—C7—H7	106.6	C1'—C6'—C7'	120.1 (2)
C6—C7—H7	106.6	N1'—C7'—C6'	112.90 (17)
C8—C7—H7	106.6	N1'—C7'—C8'	108.90 (16)
C9—C8—C7	106.97 (17)	C6'—C7'—C8'	114.75 (17)
C9—C8—C24	112.95 (17)	N1'—C7'—H7'	106.6
C7—C8—C24	114.39 (17)	C6'—C7'—H7'	106.6
C9—C8—H8	107.4	C8'—C7'—H7'	106.6
C7—C8—H8	107.4	C9'—C8'—C7'	106.51 (17)
C24—C8—H8	107.4	C9'—C8'—C24'	114.19 (18)
N3—C9—C10	126.87 (19)	C7'—C8'—C24'	113.71 (19)
N3—C9—C8	116.60 (19)	C9'—C8'—H8'	107.4
C10—C9—C8	116.37 (17)	C7'—C8'—H8'	107.4
C9—C10—C11	113.67 (17)	C24'—C8'—H8'	107.4
C9—C10—H10C	108.8	N3'—C9'—C10'	126.9 (2)
C11—C10—H10C	108.8	N3'—C9'—C8'	116.2 (2)
C9—C10—H10D	108.8	C10'—C9'—C8'	116.81 (19)
C11—C10—H10D	108.8	C9'—C10'—C11'	113.53 (18)
H10C—C10—H10D	107.7	C9'—C10'—H10A	108.9
N1—C11—C12	113.53 (16)	C11'—C10'—H10A	108.9
N1—C11—C10	111.00 (16)	C9'—C10'—H10B	108.9
C12—C11—C10	109.06 (16)	C11'—C10'—H10B	108.9
N1—C11—H11	107.7	H10A—C10'—H10B	107.7
C12—C11—H11	107.7	N1'—C11'—C12'	113.42 (16)
C10—C11—H11	107.7	N1'—C11'—C10'	111.21 (16)
C17—C12—C13	119.0 (2)	C12'—C11'—C10'	109.08 (16)
C17—C12—C11	119.2 (2)	N1'—C11'—H11'	107.6
C13—C12—C11	121.78 (19)	C12'—C11'—H11'	107.6
C14—C13—C12	120.0 (2)	C10'—C11'—H11'	107.6
C14—C13—H13	120.0	C17'—C12'—C13'	118.3 (2)
C12—C13—H13	120.0	C17'—C12'—C11'	119.51 (19)
C15—C14—C13	120.9 (3)	C13'—C12'—C11'	122.16 (18)
C15—C14—H14	119.6	C14'—C13'—C12'	120.4 (2)
C13—C14—H14	119.6	C14'—C13'—H13'	119.8
C14—C15—C16	119.8 (2)	C12'—C13'—H13'	119.8
C14—C15—H15	120.1	C15'—C14'—C13'	120.8 (2)
C16—C15—H15	120.1	C15'—C14'—H14'	119.6
C15—C16—C17	120.2 (3)	C13'—C14'—H14'	119.6
C15—C16—H16	119.9	C16'—C15'—C14'	119.5 (2)
C17—C16—H16	119.9	C16'—C15'—H15'	120.3
C12—C17—C16	120.1 (2)	C14'—C15'—H15'	120.3
C12—C17—H17	120.0	C15'—C16'—C17'	120.3 (2)
C16—C17—H17	120.0	C15'—C16'—H16'	119.9
N2—C18—N1	117.20 (17)	C17'—C16'—H16'	119.9
N2—C18—S1	120.64 (16)	C12'—C17'—C16'	120.7 (2)
N1—C18—S1	122.16 (16)	C12'—C17'—H17'	119.6
N2—C19—C20	114.7 (7)	C16'—C17'—H17'	119.6
N2—C19—H19A	108.6	N2'—C18'—N1'	116.93 (19)
C20—C19—H19A	108.6	N2'—C18'—S1'	120.93 (17)
N2—C19—H19B	108.6	N1'—C18'—S1'	122.13 (16)
C20—C19—H19B	108.6	N2'—C19'—C20'	115.6 (3)
H19A—C19—H19B	107.6	N2'—C19'—H19E	108.4
C21—C20—C19	128.1 (9)	C20'—C19'—H19E	108.4
C21—C20—H20A	115.9	N2'—C19'—H19F	108.4
C19—C20—H20A	115.9	C20'—C19'—H19F	108.4
C20—C21—H21A	120.0	H19E—C19'—H19F	107.4
C20—C21—H21B	120.0	C21'—C20'—C19'	130.6 (5)
H21A—C21—H21B	120.0	C21B—C20'—C19'	157.3 (15)
N2—C19A—C20A	111.5 (10)	C21'—C20'—H20E	114.7
N2—C19A—H19C	109.3	C19'—C20'—H20E	114.7
C20A—C19A—H19C	109.3	C21B—C20'—H20F	101.3
N2—C19A—H19D	109.3	C19'—C20'—H20F	101.3
C20A—C19A—H19D	109.3	C20'—C21'—H21E	120.0
H19C—C19A—H19D	108.0	C20'—C21'—H21F	120.0
C21A—C20A—C19A	126.9 (17)	H21E—C21'—H21F	120.0
C21A—C20A—H20C	116.6	C20'—C21B—H21G	120.0
C19A—C20A—H20C	116.6	C20'—C21B—H21H	120.0
C20A—C21A—H21C	120.0	H21G—C21B—H21H	120.0
C20A—C21A—H21D	120.0	O2'—C22'—O1'	116.2 (3)
H21C—C21A—H21D	120.0	O2'—C22'—C23'	126.3 (3)
O2—C22—O1	116.7 (3)	O1'—C22'—C23'	117.4 (3)
O2—C22—C23	125.3 (2)	C22'—C23'—H23D	109.5
O1—C22—C23	118.0 (2)	C22'—C23'—H23E	109.5
C22—C23—H23A	109.5	H23D—C23'—H23E	109.5
C22—C23—H23B	109.5	C22'—C23'—H23F	109.5
H23A—C23—H23B	109.5	H23D—C23'—H23F	109.5
C22—C23—H23C	109.5	H23E—C23'—H23F	109.5
H23A—C23—H23C	109.5	C25'—C24'—C26'	110.1 (2)
H23B—C23—H23C	109.5	C25'—C24'—C8'	110.5 (2)
C25—C24—C26	110.5 (2)	C26'—C24'—C8'	111.3 (2)
C25—C24—C8	110.79 (19)	C25'—C24'—H24'	108.3
C26—C24—C8	111.3 (2)	C26'—C24'—H24'	108.3
C25—C24—H24	108.0	C8'—C24'—H24'	108.3
C26—C24—H24	108.0	C24'—C25'—H25D	109.5
C8—C24—H24	108.0	C24'—C25'—H25E	109.5
C24—C25—H25A	109.5	H25D—C25'—H25E	109.5
C24—C25—H25B	109.5	C24'—C25'—H25F	109.5
H25A—C25—H25B	109.5	H25D—C25'—H25F	109.5
C24—C25—H25C	109.5	H25E—C25'—H25F	109.5
H25A—C25—H25C	109.5	C24'—C26'—H26D	109.5
H25B—C25—H25C	109.5	C24'—C26'—H26E	109.5
C24—C26—H26A	109.5	H26D—C26'—H26E	109.5
C24—C26—H26B	109.5	C24'—C26'—H26F	109.5
H26A—C26—H26B	109.5	H26D—C26'—H26F	109.5
C24—C26—H26C	109.5	H26E—C26'—H26F	109.5
H26A—C26—H26C	109.5	C18'—N1'—C7'	118.81 (17)
H26B—C26—H26C	109.5	C18'—N1'—C11'	121.76 (16)
C18—N1—C7	118.77 (16)	C7'—N1'—C11'	116.86 (16)
C18—N1—C11	121.97 (16)	C18'—N2'—C19'	125.3 (2)
C7—N1—C11	116.89 (15)	C18'—N2'—H2B	121.5 (16)
C18—N2—C19	125.1 (8)	C19'—N2'—H2B	112.1 (16)
C18—N2—C19A	125.4 (13)	C9'—N3'—O1'	110.08 (18)
C18—N2—H2A	120.1 (16)	C22'—O1'—N3'	113.1 (2)

### Hydrogen-bond geometry (Å, º)

**Table:**

*D*—H···*A*	*D*—H	H···*A*	*D*···*A*	*D*—H···*A*
C2—H2···O2^i^	0.93	2.59	3.298 (4)	133
C2′—H2′···O2′^ii^	0.93	2.59	3.332 (4)	137

Symmetry codes: (i) −*x*+2, −*y*+1, −*z*; (ii) −*x*+1, −*y*+2, −*z*+1.
