# Supplementary figures and images for: Crystal structure of (E)-4-(acet­oxy­imino)-N-allyl-3-isopropyl-2,6-di­phenyl­piperi­dine-1-carbo­thio­amide
Source: Acta Crystallogr E Crystallogr Commun. 2015 Jul 4;71(Pt 8):o542–3. doi: 10.1107/S2056989015012499 (PMC4571387; doi:10.1107/S2056989015012499)

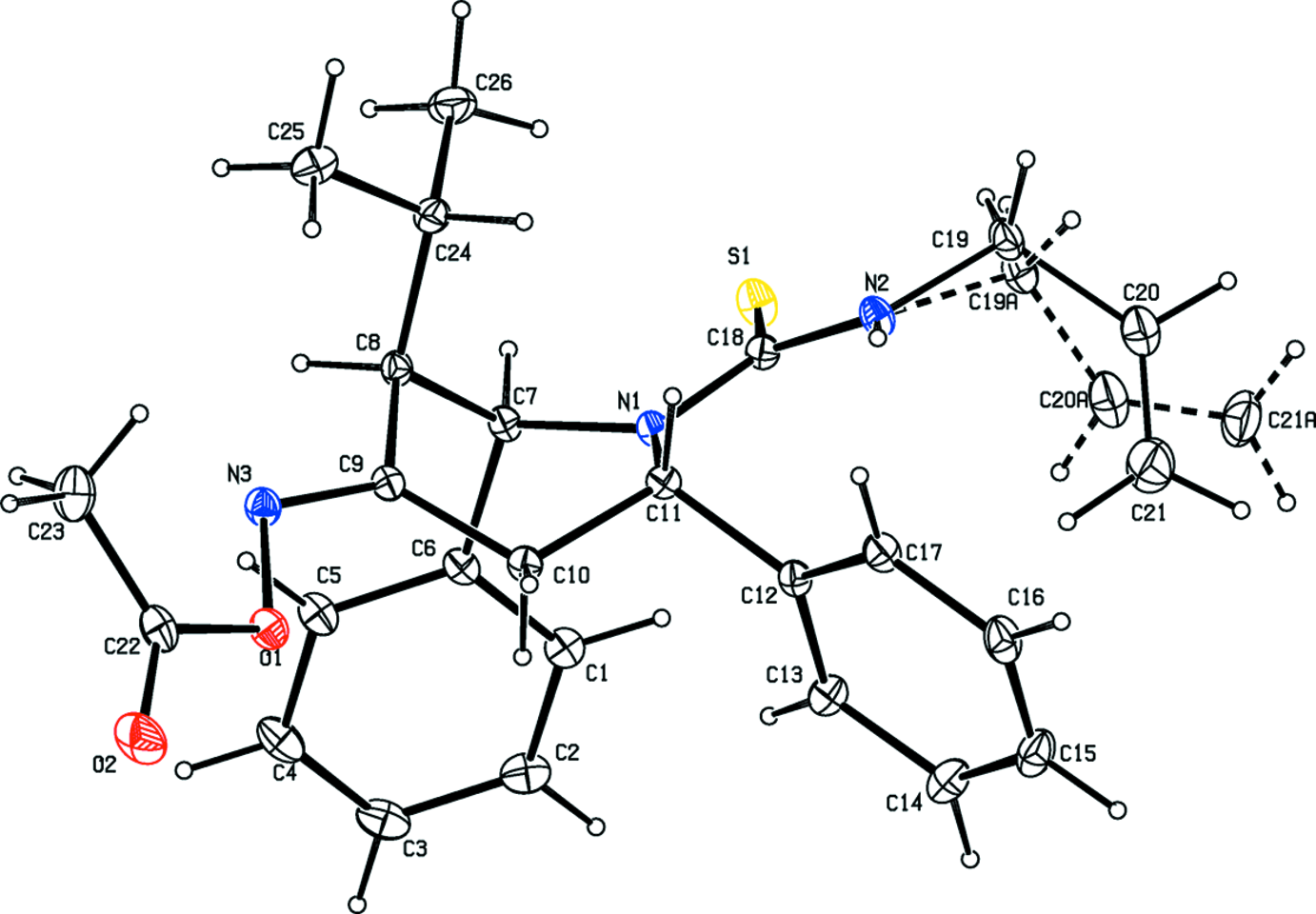

Supplement: Supplementary file 4 [file e-71-0o542-fig1.tif]

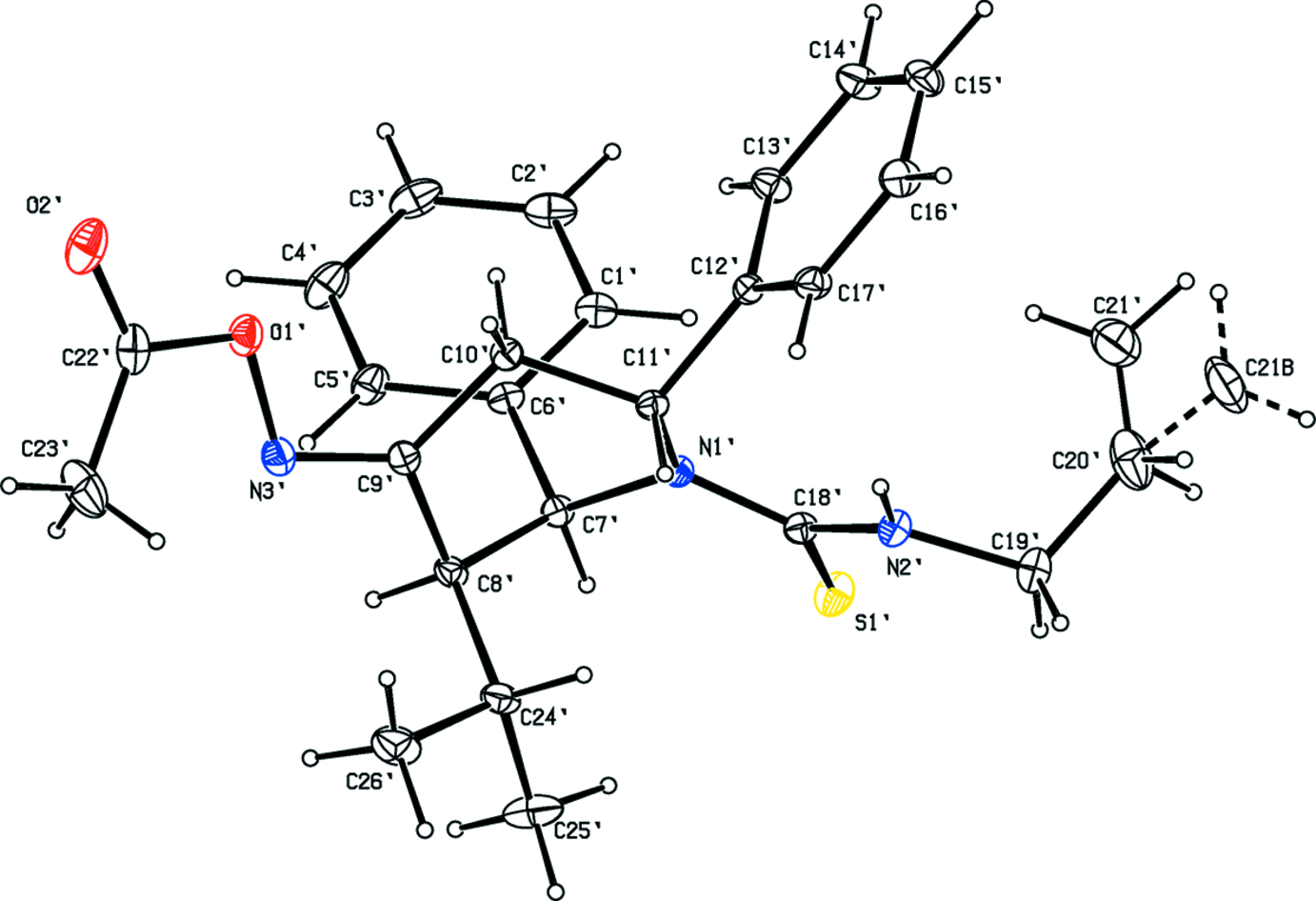

Supplement: Supplementary file 5 [file e-71-0o542-fig2.tif]

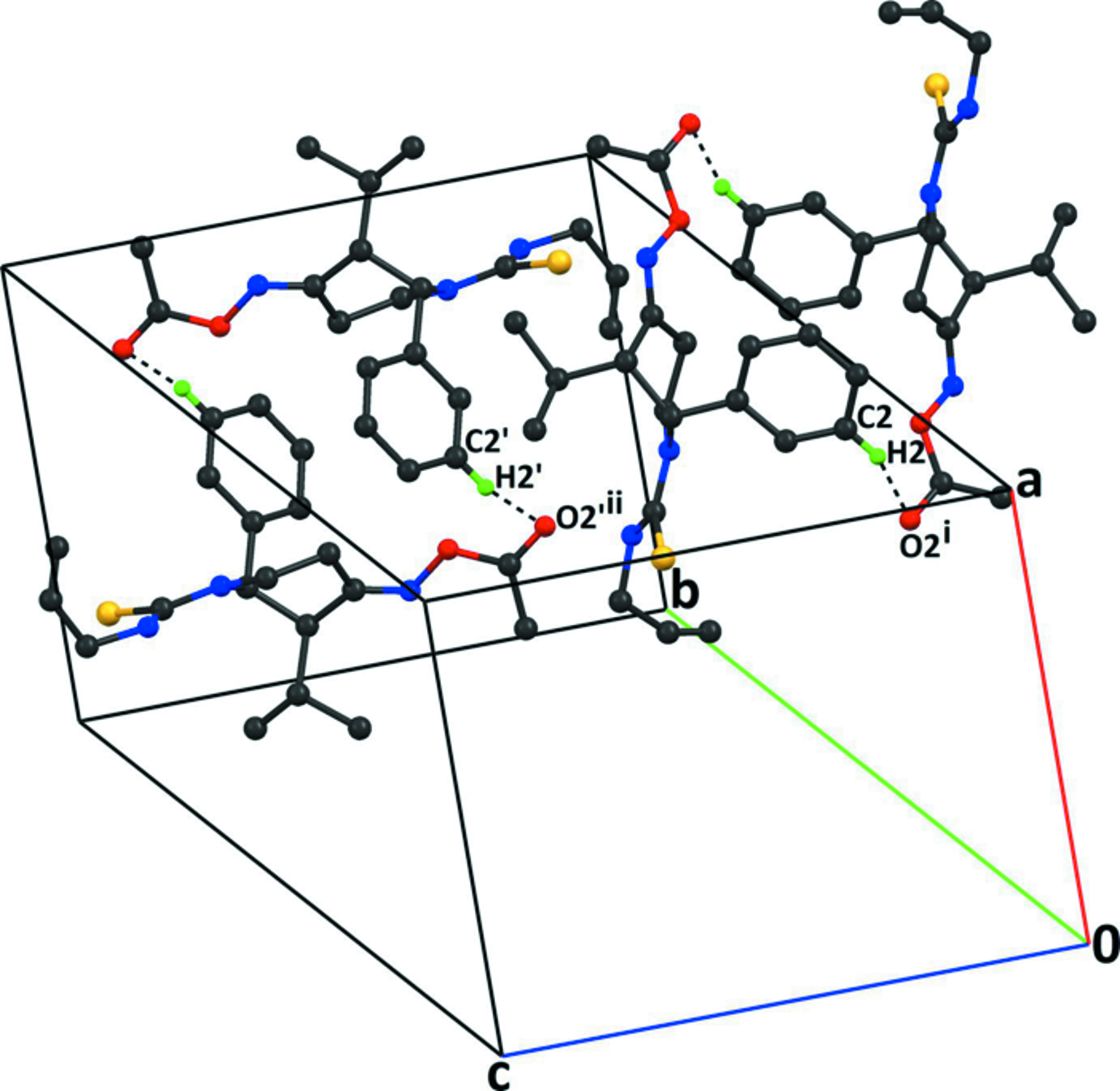

Supplement: Supplementary file 6 [file e-71-0o542-fig3.tif]
